# Alterations in muscle mass and contractile phenotype in response to unloading models: role of transcriptional/pretranslational mechanisms

**DOI:** 10.3389/fphys.2013.00284

**Published:** 2013-10-11

**Authors:** Kenneth M. Baldwin, Fadia Haddad, Clay E. Pandorf, Roland R. Roy, V. Reggie Edgerton

**Affiliations:** ^1^Department of Physiology and Biophysics, University of California, Irvine, IrvineCA, USA; ^2^Department of Pediatrics, Pediatric Exercise Research Center, University of California, Irvine, IrvineCA, USA; ^3^Division of Basic Medical Sciences, Mercer University School of Medicine, MaconGA, USA; ^4^Departments of Integrative Biology and Physiology, University of California, Los Angeles, Los AngelesCA, USA; ^5^Brain Research Institute, University of California, Los Angeles, Los AngelesCA, USA; ^6^Departments of Neurosurgery, University of California, Los Angeles, Los AngelesCA, USA; ^7^Departments of Neurobiology, University of California, Los Angeles, Los AngelesCA, USA

**Keywords:** spaceflight, hindlimb suspension (unloading), spinal cord isolation, myosin isoforms, non-coding RNAs

## Abstract

Skeletal muscle is the largest organ system in mammalian organisms providing postural control and movement patterns of varying intensity. Through evolution, skeletal muscle fibers have evolved into three phenotype clusters defined as a motor unit which consists of all muscle fibers innervated by a single motoneuron linking varying numbers of fibers of similar phenotype. This fundamental organization of the motor unit reflects the fact that there is a remarkable interdependence of gene regulation between the motoneurons and the muscle mainly via activity-dependent mechanisms. These fiber types can be classified via the primary type of myosin heavy chain (MHC) gene expressed in the motor unit. Four MHC gene encoded proteins have been identified in striated muscle: slow type I MHC and three fast MHC types, IIa, IIx, and IIb. These MHCs dictate the intrinsic contraction speed of the myofiber with the type I generating the slowest and IIb the fastest contractile speed. Over the last ~35 years, a large body of knowledge suggests that altered loading state cause both fiber atrophy/wasting and a slow to fast shift in the contractile phenotype in the target muscle(s). Hence, this review will examine findings from three different animal models of unloading: (1) space flight (SF), i.e., microgravity; (2) hindlimb suspension (HS), a procedure that chronically eliminates weight bearing of the lower limbs; and (3) spinal cord isolation (SI), a surgical procedure that eliminates neural activation of the motoneurons and associated muscles while maintaining neurotrophic motoneuron-muscle connectivity. The collective findings demonstrate: (1) all three models show a similar pattern of fiber atrophy with differences mainly in the magnitude and kinetics of alteration; (2) transcriptional/pretranslational processes play a major role in both the atrophy process and phenotype shifts; and (3) signaling pathways impacting these alterations appear to be similar in each of the models investigated.

## Introduction

**Figure 1:**
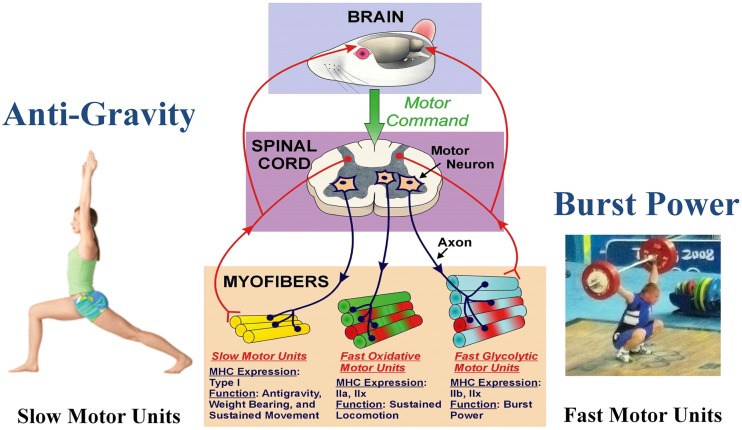
**Functional specialization of skeletal muscle**. The brain initiates the motor command for a motor neuron to fire and stimulate a group of muscle fibers to contract. A motor unit consists of a single motor neuron together with all the muscle fibers it innervates. Different types of motor units express different MHC phenotypes, having a specialized function. Note that each myofiber can express either a single MHC isoform, or a hybrid mix of two or more isoforms. Slow motor unit expresses type I and are engaged in antigravity postures. The fast glycolytic motor units express IIb and IIx, and are recruited during burst power like during weight lifting. (Weight lifting image is Wikimedia Commons depicting Andrei Rybakou of Belarus Weightlifting at the 2008 Summer Olympics in the 85 kg category. This image is licensed under the Creative Commons Attribution 2.0 Generic license).

Skeletal muscle is the largest organ system in all mammals, including humans. This integrated system consists of hundreds of individual muscles, which provide postural control during upright posture (e.g., standing) along with a wide range of movement patterns of varying intensity performed under various loading conditions imposed by the force of gravity. Through evolution in the gravity environment, skeletal muscle fibers have evolved into essentially three generic phenotype clusters defined as a motor unit. The motor unit consists of a motoneuron and all of the muscle fibers innervated by that motoneuron (Edström and Kugelberg, 1968; Burke et al., 1971). Through multiple mechanisms, a major one being activity-dependency, those fibers in a given motor unit express a similar metabolic/contractile phenotype. As presented in Figure 1, these fiber types can be classified as slow-oxidative, fast-oxidative-glycolytic, and fast-glycolytic (Peter et al., 1972). The inherent contractile speed of each fiber-type cluster is determined essentially by the myosin motor protein isoform predominantly expressed. For example, the slow-oxidative unit expresses primarily a slow myosin heavy chain (MHC) gene designated as slow, type I. The fast-oxidative unit expresses a combination of the fast type IIa and IIx MHC genes, whereas the fast-glycolytic unit expresses a combination of the fast IIb and IIx MHC genes (Larsson et al., 1991). Concerning the fast MHC isoforms, humans generally do not express the fast type IIb isoform at the protein level, whereas the IIb isoform is highly expressed in the limb muscles [e.g., vastus lateralis (VL), gastrocnemius, and plantaris] of small animals such as rodents (Booth and Baldwin, 1996).

During the last 40 years, investigators have generated considerable information demonstrating the powerful control that altered loading states (e.g., alterations in weight bearing activity opposing gravity) play in modulating muscle fiber size as well as the contractile and metabolic phenotypes in the different types of motor units, especially those expressing an abundance of the slow-type I and type IIa MHCs. This information has been gathered to a large extent via studies involving small animal models such as the rat in response to interventions such as space flight/microgravity, hindlimb unloading via hindlimb suspension (HS), and the novel model of “spinal cord isolation” (SI) whereby the target neuromuscular units are inactivated while maintaining an intact motoneuron-to-muscle fiber connection (Roy et al., 1991, 1996).

The primary goal of this review is to examine the putative mechanisms that cause both muscle atrophy and alterations in the contractile phenotype by integrating what has been learned from these three unique inactivity/unloading paradigms. The information to be presented is certainly relevant to similar alterations that have been observed in human skeletal muscles (Booth and Baldwin, 1996). However, due to page limitations for this review series, we will focus our attention primarily on the rat model.

## Relevant fundamental concepts

### Muscle plasticity

Skeletal muscle is unique in that its structural, contractile, and metabolic properties are sensitive to the various demands imposed on the muscular systems, especially the lower limb muscles which bear the brunt of opposing gravity and carrying out movement activities of varying intensity and duration. For example, if one performs aerobic exercise, such as distance running on a regular basis, the mitochondrial system within the fibers undergoes *de novo* biogenesis to increase the number of mitochondria to enhance the duration that the muscles can function without fatiguing. However, the muscle fibers engaged in this type of activity do not hypertrophy (Booth and Baldwin, 1996). On the other hand, if one performs high loading resistance exercise (RE), the muscle fibers increase their cross sectional area by increasing abundance of the contractile machinery without necessarily increasing the mitochondrial density. Thus, the various sub-cellular components of the muscle fiber adapt to the nature of the stimulus, or lack thereof. In this review, we will focus on environmental factors that reduce mechanical and metabolic stress on the muscles rather than adding more stress to enhance the functional properties of the fibers.

### Muscle protein turnover

**Figure 2:**
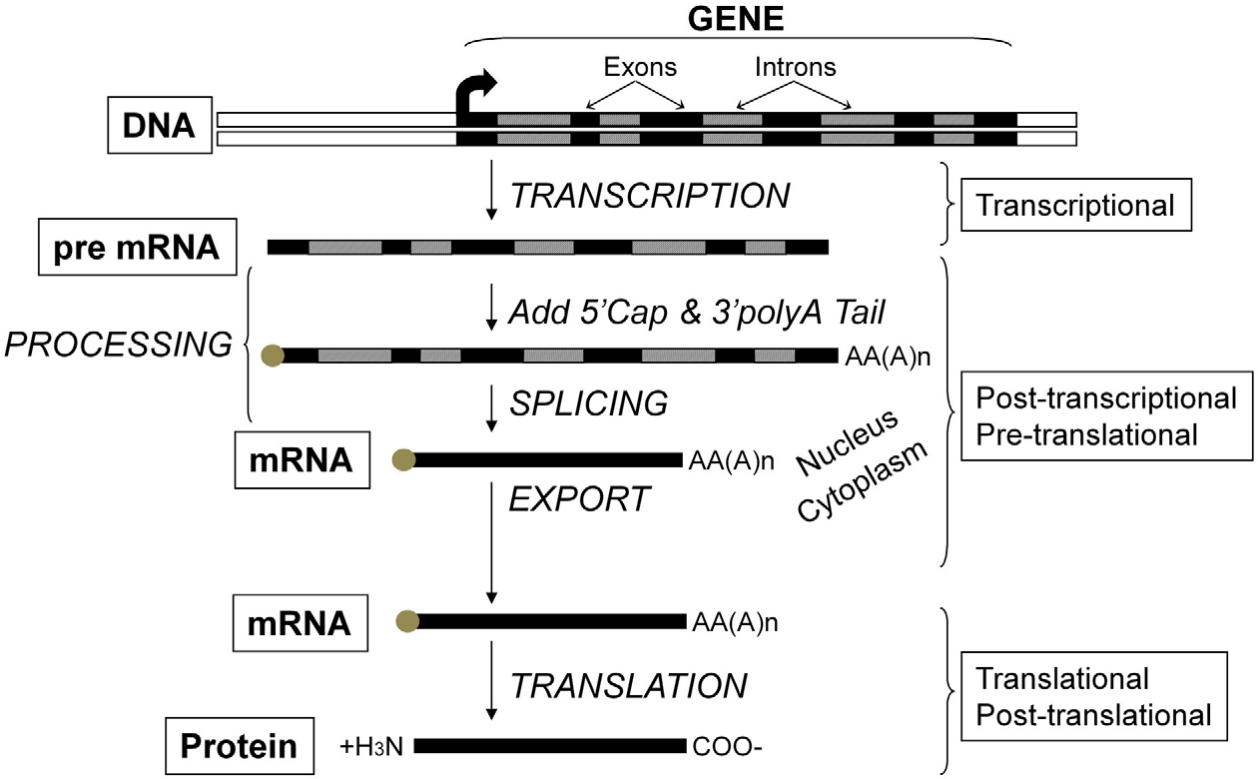
**Flow of genetic information and key steps in the regulation of gene expression**. The level of protein expressed in the cell results from the net balance between protein synthesis and protein degradation. Protein synthesis can be regulated via several processes including those operating at the transcriptional, post-transcriptional, pre-translational, translational, and post-translational levels. The product of each step is subjected to degradation control.

**Figure 3:**
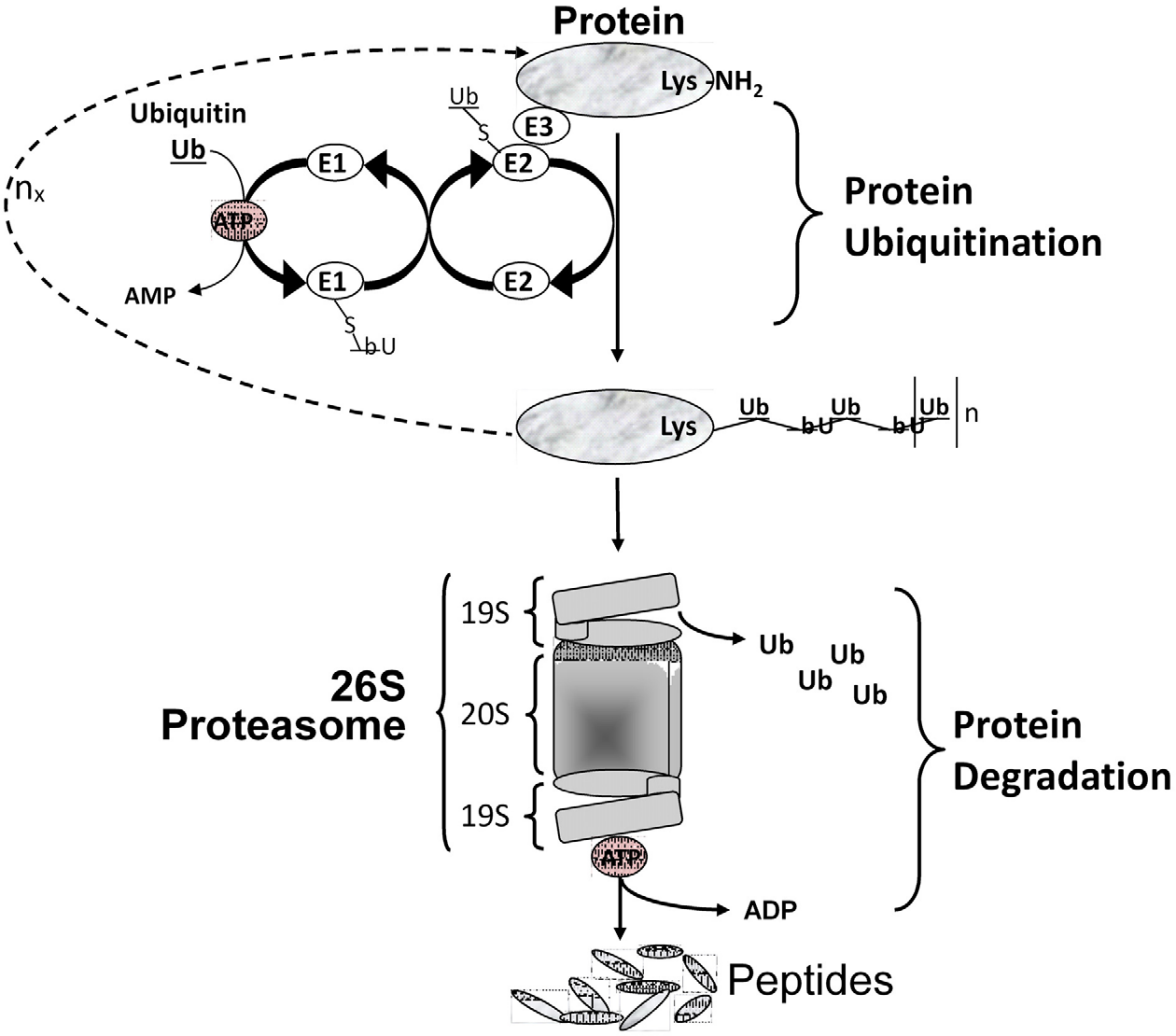
**Protein degradation via the ubiquitin/proteasome system**. Multiple Ub molecules are covalently conjugated to amino groups of the protein. Ub is first activated by E1 (Ub activating enzyme), then transferred to E2 (Ub carrier protein or Ub-conjugating enzyme) to be ligated to an amino group of the target protein with the help of E3 (Ub-protein ligase). This is followed by a sequential conjugation of additional Ub molecules each linked to an NH2 group of a lysine of the previously added Ub, thereby generating a polyubiquitinated protein that becomes recognized by the 26S proteasome machinery and targeted for degradation.

Critical to the concept of muscle plasticity noted above involves the phenomenon that the various proteins comprising skeletal muscle fibers are continually turning over. As presented in Figure 2, any given gene can undergo altered expression via the genomic process of transcription thereby producing a pre-mRNA transcript that serves as the primary RNA product. This transcriptional product then is altered in several ways during transformation into mature mRNA, thus becoming the blueprint for translation into the protein product (Figure 2). This phase is also referred to as protein synthesis/translation. Subsequently, the protein becomes targeted for degradation largely via the ubiquitin-proteasome pathway that involves N-end rule as depicted in Figure 3. Since the contractile apparatus (i.e., the myofibril fraction) is the key functional component of the muscle fiber and accounts for ~50–60% of the total protein expressed in the muscle cell, we will focus on this particular system in this review.

Multiple intracellular proteolytic schemes exist in skeletal muscle including the lysosomal pathway, calcium-activated proteases (calpains), and the ATP-dependent system that involves the ubiquitin-proteasome pathway. This latter is responsible for most of myofibrillar protein degradation in various forms of muscle atrophy and wasting (Solomon et al., 1998). To degrade the contractile proteins comprising the myofibril pool, three sequential events must occur. Firstly, the myofibril machinery must undergo the initial process of proteolysis to disassemble the contractile machinery. This process is thought to be regulated by calcium activated proteolytic enzymes such as the calpains and caspases (Goll et al., 2008). Secondly, these naked proteins become targeted by the process of “ubiquitin-conjugation” which occurs largely through the N-end rule pathway involving a three-enzyme-step reaction involving ligation of the target protein with poly-ubiquitin molecules (Figure 3). It is known that specific E3 ligase isoforms are responsible for the specificity of targeting any given protein for destruction (Sacheck et al., 2007). Thirdly, once ubiquitinated the target protein is transported to the 26S proteasome complex located in the cytosol where the protein is progressively broken down into small peptides and eventually to free amino acids, the latter of which can be recycled. As depicted in Figure 3, this latter degradation step is highly dependent on ATP as the energy source.

The mechanisms governing each step of the end-to-end process of protein turnover has been examined in considerable detail (Goll et al., 2008; Rasmussen and Richter, 2009). In examining the concept of muscle fiber protein homeostasis, it follows that the size of any given muscle fiber is predicated on the ratio of protein translation activity relative to protein degradation activity. During states in which fiber hypertrophy is occurring, the relative balance favors a net synthesis capacity relative to the process of degradation, whereas when the muscle is atrophying the balance is skewed to greater degradation relative to synthesis.

## Responsiveness of different muscle types to unloading in various models

### Spaceflight/microgravity

**Figure 4:**
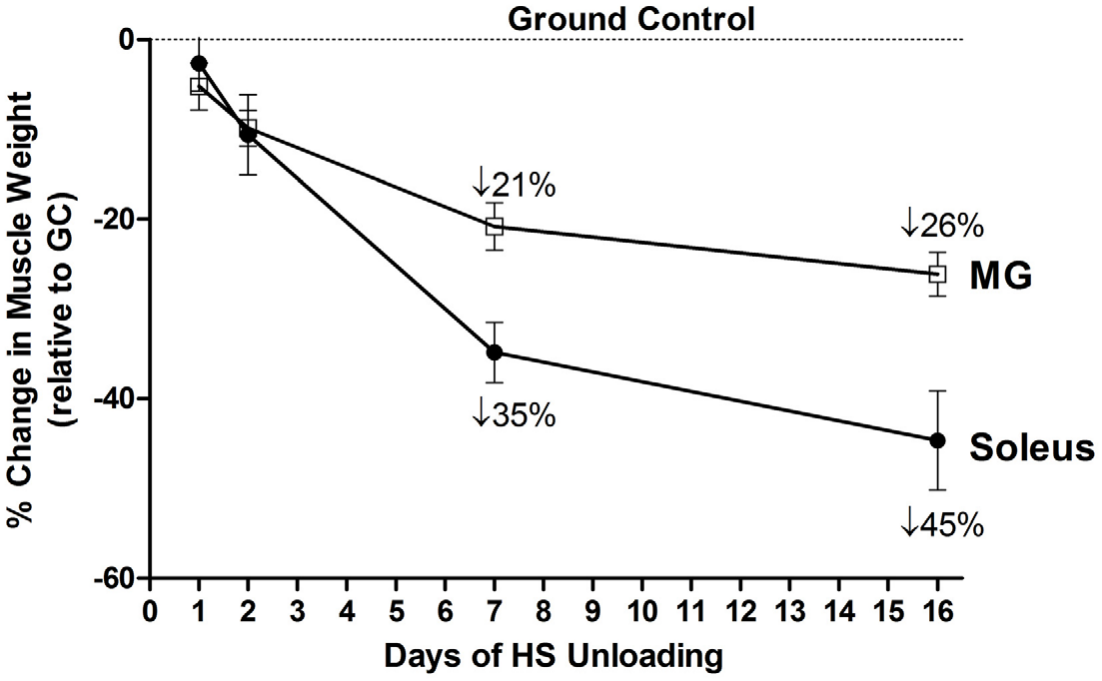
**Both soleus and medial gastrocnemius (MG) atrophy in response to unloading**. Change in muscle mass relative to control of both MG and Soleus in response to 16 days of hindlimb suspension in rats.

The environment of spaceflight/microgravity is most unique in that the ground reaction forces essentially are eliminated, and the organism is essentially in a state of “free-fall” thereby markedly reducing the forces generated by the limb and core body muscles. Initial studies sponsored by both the NASA Space Life Sciences Programs and the Soviet Cosmos Biosputnik Research. Programs were carried out from the late 1980s to ~2000. These experiments were of relatively short duration lasting between 5 and 22 days in the microgravity environment (Martin et al., 1988). Skeletal muscle investigations focused on morphological, histochemical, enzymatic, metabolic, and biochemical analyses of lower limb muscles consisting of different fiber type profiles (Figure 1) such as the triceps surae (gastrocnemius, plantaris, and soleus), the quadriceps [rectus femoris, vastus intermedius (VI), VL, and vastus intermedius (VI)] and the adductor longus (AL) muscle groups. It is important to note that the soleus, VI, and AL muscles are primarily comprised of slow-type I fibers, whereas the medial gastrocnemius (MG), plantaris and VL are primarily comprised of fast type II fibers. As delineated below, rodent muscles that express predominantly slow motor units appear to be more sensitive to the unloading stimuli compared to muscles expressing primarily the fast motor units as presented in Figure 4. It is interesting to note, however, that this greater responsiveness is primarily related to the predominant phenotype within a muscle more than it is to the individual fiber phenotype. For example a slow fiber in the faster MG atrophies less than the same phenotype in the slower soleus (Roy et al., 1991).

One of the first notable experiments involved the 12.5 days of microgravity exposure on the Biosatellite Cosmos 1887, which was flown in 1987 (Miu et al., 1989; Baldwin et al., 1990). A key finding from this mission was a significant loss (~25%) of muscle mass in both the VI and soleus muscles. Analyses of myosin isoforms in the myofibril fraction indicated that slow type I MHC was the primary isoform lost in the calculated degradation of total MHC protein complex. Additional findings (Martin et al., 1988; Miu et al., 1989) also demonstrated a “*de novo*” expression of the fast type II MHC isoforms in the slow soleus muscle, indicating a switching of MHC isoform expression in the atrophied soleus muscle.

This foundation of information was amplified further by a series of NASA's integrated Space Life Sciences Missions which began in 1991. It is important to note that these particular missions were actually preceded by a pilot mission carried out in 1985 on the NASA space shuttle to validate the housing configuration for future rodent studies (Martin et al., 1988). For example, as reported by Haddad et al. (1993) following the 9 day Space Lab 1 Mission, the VI muscle was atrophied by ~22% after the 9 day Space Lab 1 Mission. This atrophy was associated with a loss in type I and fast type, IIa MHC isoforms, which were counteracted by increases in expression of the faster type IIx and IIb isoforms. Importantly, these findings provided a biochemical analysis to corroborate the histochemical findings generated by Martin et al. on the NASA space shuttle 7-day SL-3 mission reported above (Martin et al., 1988). Furthermore, the investigation by Haddad et al. (1993) also was one of the first reports to demonstrate alterations in mRNA expression in the target muscles. These results complemented the findings at the protein level and suggested that molecular alterations involving both pretranslational and translational events were involved in MHC gene regulation during these unloading paradigms.

In addition to the above findings, two additional relevant NASA Space Life Science Missions were carried out in the 1990s. The first was the 6-day mission STS-54 that was designed to correlate alterations in muscle phenotype with intrinsic functional properties of the soleus muscle (Caiozzo et al., 1994). The key findings demonstrated that microgravity induced a rapid decrease in soleus muscle mass (27% within 6 days) along with a shift from slow to fast MHC phenotypes that was correlated with a shift in the force-velocity properties of the soleus muscle. In effect, the soleus became weaker, faster, and more fatigable after the flight.

The second study was the 14 day mission STS-58 (Space Lab Life Sciences 2) that demonstrated a further reduction in muscle mass relative to body weight (34%) and a greater shift in MHC expression from a slow to fast isoform pattern (Caiozzo et al., 1996). Furthermore, monoclonal histochemical analyses of individual muscle fibers demonstrated a significant expression of hybrid fibers containing both slow and fast MHC isoforms. It also became apparent that the alterations in MHC expression favored a greater transformation to the faster type IIx and IIb MHC isoforms the longer the exposure to microgravity. In addition, mRNA responses were more robust than the changes in the corresponding protein isoforms suggesting that further studies were necessary to better integrate transcriptional, pre-translational, and post-translational processes.

In the context of the above findings, it is important to point out that a major drawback with the spaceflight missions was the short duration of the missions carried out to date with little likelihood that longer duration missions could be expanded until facilities with animal habitats could be provided on the International Space Station (ISS) in 2000. Unfortunately, these habitats were never developed due to insufficient resources provided by the different International Space Programs supporting research on the ISS. Thus, it became apparent that other models mimicking the space flight environment with regard to altered loading state and muscle function were necessary to expand the knowledge base pertaining to physiological alterations in skeletal muscle.

### Hindlimb suspension

During the same time frame that the Space Shuttle/Biosputnik Missions were taking place, investigators also began experimenting with a ground-based unloading model for comparison with the space flight studies. This model consisted of applying traction to the tail of the adult rat in order to lift the hind legs off the ground thereby eliminating ground reactions forces impacting the homeostasis of the limb muscles (Musacchia et al., 1983; Jaspers et al., 1985; Fitts et al., 1986; Thomason et al., 1987).

The HS study by Thomason et al. is noteworthy in that a 56-day time course study was carried out to examine changes in muscle mass, myofibril content, and slow/fast MHC isoform content (Thomason et al., 1987). The key findings of this study were that by 16 days of unloading there was a ~50% atrophy in the slow soleus, VI, and AL muscles when normalized to body weight. This loss in muscle mass was attributed to a net degradation of the myofibril protein fraction, especially the slow MHC protein component of the slow muscle fibers, based on quantitative MHC analyses of the soleus muscle. It is important to point out that the degree of muscle atrophy that occurred during the early stages of unloading in this model was equivalent to what was observed in the space flight missions of similar duration as presented above. Since the effects of HS on the mass of fast muscles such as the plantaris and MG were not as robust as that for the slow muscle types (Figure 4), it is apparent that muscles expressing predominately the slow MHC isoform in rodents are more sensitive to reduced weight-bearing activity.

In the context of these findings, it has been demonstrated that hyperthyroidism (i.e., T3 treatment) also induces a marked down regulation of slow, type I MHC gene expression while concomitantly increasing expression of the type II MHC genes without necessarily inducing muscle atrophy (Caiozzo et al., 1991, 1993). These observations raised the question as to whether both unloading (HS) and T3 treatment cause slow type I MHC repression via a common process/pathway. To address this issue, a series of experiments were conducted to examine both the separate and combined effects of HS and T3 treatment on MHC isoform expression spanning a time interval of 28 days (Caiozzo et al., 1997, 1998). The key findings of these experiments demonstrated that both T3 treatment and HS individually induced a ~40–50% loss in slow type I MHC expression at both the mRNA and protein levels. In contrast, when the two interventions were combined, all slow type I fibers were induced to express various combinations of fast type II MHC isoforms, i.e., types IIa, IIx, and IIb. These hybrid MHC fibers included novel combinations such as I/IIa/IIx, I/IIx/IIb, and I/IIa/IIx/IIb (Caiozzo et al., 1998). Thus, these findings demonstrated that the soleus muscle does not necessarily contain refractory fibers in response to unloading. Rather, this slow muscle fiber type contains different populations of slow fibers that vary in their sensitivity to various altered physiological conditions such as thyroid state and mechanical loading stimuli. Alternatively, it may be that the combination of these mechanical, neural, and metabolic perturbations ablate all sources of fast and slow phenotype protein regulation that normally exists, thus eliminating, at least temporarily, all of the crucial mechanisms normally underlying muscle phenotypes.

### Spinal cord isolation

While the models of spaceflight and of hind limb suspension induce marked alterations in both MHC gene expression and muscle fiber mass compared to normal weight-bearing rats, it is difficult to quantify the amount of neuromuscular activation that is necessary for these target muscles to maintain their homeostasis in response to these contrasting types of interventions. One model that addresses this dilemma is the unique model of SI in which the limb muscle fibers are neurologically silenced while maintaining an intact innervation for prolonged periods (Pierotti et al., 1991; Roy et al., 1992). In this model, the lumbar region of the spinal cord is functionally isolated by complete spinal cord transection at both the cervical and sacral levels, along with performing a bilateral dorsal rhizotomy between the two transection sites. This surgical procedure eliminates supraspinal, infraspinal, and peripheral afferent input to the spinal cord segments while leaving the motoneuron-muscle fiber connection intact. Thus, SI provides a model that removes both neuromuscular activation and loading stimuli while the motoneurons continue to exert activity-independent neurotrophic effects on the inactive muscle fibers (Roy et al., 1992).

In 2001, our research group conducted a longitudinal study spanning 90 days in which SI rats were compared to their weight bearing counterparts after 4, 8, 15, 30, 60, and 90 days of SI (Huey et al., 2001). The mean soleus muscle to body weight ratios (mg/gram) were reduced by 10, 27, 43, 53, 55, and 66%, respectively over these time points indicating that the neuromuscular inactivation (including non-weight-bearing activity) resulted in a marked degree of atrophy in the target soleus muscle. Beginning on day 15 and up to 90 days of SI, the type I MHC mRNA expression was significantly decreased; whereas, MHC protein did not significantly decrease until day 30 and 60 in both the slow soleus and AL muscles. However, in both muscles, slow MHC down regulation was offset by significant up regulation of the faster type II MHC isoforms, especially the IIx MHC. From 60 to 90 days of intervention, the type I MHC was almost completely replaced with the faster type II isoforms when examined at both the mRNA and protein levels of analyses. Thus, in the SI model chronic inactivity and unloading of slow rat hindlimb muscles shifted their MHC profile from predominantly type I to type IIx mRNA and protein. Based on this study, additional studies were performed to gain more insight concerning the transcriptional/pretranslational mechanisms involved in this model, as presented below.

## A chronological assessment of transcriptional/pretranslational mechanisms impacting muscle mass and contractile phenotype in animal models of chronic unloading

### Spinal cord isolation model

Given the rapid loss in muscle mass along with the marked shift over time from a slow to fast muscle contractile phenotype in response to SI at both the mRNA and protein level, our integrated group performed a series of experiments to characterize both the molecular and cellular processes linked to these alterations (Huey et al., 2002; Haddad et al., 2003a,b).

First of all, we tested the hypothesis that the down regulation of type I MHC expression in the soleus muscle of SI rats is regulated at the transcriptional level by using the *in vivo* direct gene transfer approach to identify key regulatory elements in the type I MHC promoter responsible for the inhibition of type I MHC gene transcription (Huey et al., 2002). To perform these experiments, we first validated the *in vivo* gene injection technique in rats, which is the animal model we used for all of our transcriptional mechanistic studies (Giger et al., 2000). With this direct gene transfer approach, we determined the activity of different length type I MHC promoter fragments, linked to a firefly luciferase reporter gene in soleus muscle of control and SI rats. One week of SI significantly decreased *in vivo* activity of the −3500, −408, −299, −215, and −171 base pair (bp) type I MHC promoter fragments. When the activity of all the tested promoters were expressed relative to activity of the skeletal actin promoter (to normalize the data) all of the slow MHC promoters tested were significantly reduced in the SI soleus except the short −171 bp promoter, which was significantly elevated. Mutation of the βe3 element (−214/190 bp) in both the −215 and −408-bp promoters and deletion of this element in the −171-bp promoter attenuated type I down regulation in response to SI. Also, gel mobility shift assays demonstrated a decrease in the transcription enhance factor-1 (TEF-1) binding to the βe3 element in response to SI. These results indicated that the type I MHC down regulation with SI is indeed regulated at the transcriptional level. Also, our findings suggested that interactions between the TEF-1 transcription factor and the βe3 element were likely pivotal to this response.

Additional studies focused on time-course quantitation of myosin expression at both the protein and mRNA level in response to SI (Haddad et al., 2003a,b). Adult female rats were assigned randomly to normal control and SI groups and then studied at 0, 2, 4, 8, and 15 days following SI surgery. The slow soleus muscle atrophied by 50% at the 15 day time point with the greatest loss occurring within the first 8 days. The concentration of myofibril protein steadily decreased between 4 and 15 days of SI, and this alteration was associated with a 50% decrease in MHC protein normalized to the total protein content. The concentration of actin, relative to total protein was impacted to a lesser extent. Interestingly, marked reductions occurred in total RNA (of which ~85% is ribosomal) along with a decrease in DNA content. Also, total MHC and actin mRNA expressed relative to 18S ribosomal RNA was markedly reduced consistent with the promoter studies presented above.

These findings suggest that two key factors contributed to the muscle atrophy that occurs in the SI model: (1) total ribosomal RNA concentration is reduced, which results in a reduction in “protein translational capacity”; and (2) insufficient mRNA substrate is maintained for the translation of key sarcomeric proteins comprising the myofibril fraction such as MHC and actin. In addition, the marked selective depletion of MHC protein in the muscles of the SI rats suggests that this protein (MHC) is more sensitive to inactivity than the actin, even though the actin protein content also is significantly decreased. Collectively, these data are consistent with the involvement of both transcriptional/pretranslational and translational processes contributing to the marked muscle atrophy that occurs in response to SI. Furthermore, this study provided important evidence that those atrophy processes that occur in the absence of weight-bearing activities, such as chronic disuse and spaceflight, are not solely regulated by protein degradation processes as presented in Figure 3. Rather, the atrophy process is strongly influenced by events that negatively impact the muscle's ability to generate sufficient sarcomeric protein to offset the enhanced degradation process that is occurring during the early stages of unloading.

The findings noted above were complemented further by focusing on transcriptional events using semi-quantitative RT-PCR to analyze the expression of slow type I MHC along with α-skeletal actin gene expression at both the pre-mRNA and mature mRNA level in control and 8-day SI soleus RNA samples (Haddad et al., 2003b). We also examined key signaling pathway markers for both protein translation and degradation processes. SI was associated with reduced transcriptional activity (via pre-mRNA analyses for both slow type I myosin and alpha-actin). In addition, there was an increase in gene expression of those enzyme systems enhancing protein degradation (calpains), and enzymes associated with polyubiquitination processes, e.g., atrogin-1 (E3α Ub ligase) that further contribute to the protein deficits occurring in the SI muscles via the up-regulation of the degradation pathways presented in Figure 3.

Interestingly, IGF-1, IGF-1 receptor, and IGF-1 binding proteins 4 and 5 mRNA expression were markedly induced. These phenomena normally occur under conditions that turn on muscle hypertrophy (Adams et al., 2004). We speculate that these IGF-1 anabolic stimuli are most likely being turned on to counteract the enhanced elevation in protein degradation that occurs during SI (Sacheck et al., 2007). In addition, phospho-ERK-1 and -ERK-2 were elevated. Since both the IGF-1 and ERK1/2 cascades have been implicated as key signaling pathways necessary for inducing normal, as well as anabolic/hypertrophic growth processes in skeletal muscle (Adams et al., 2004), these latter observations in the SI model are consistent with anabolic stimuli being turned on to offset the degradation cascade that occurs during the early stages of SI.

**Figure 5:**
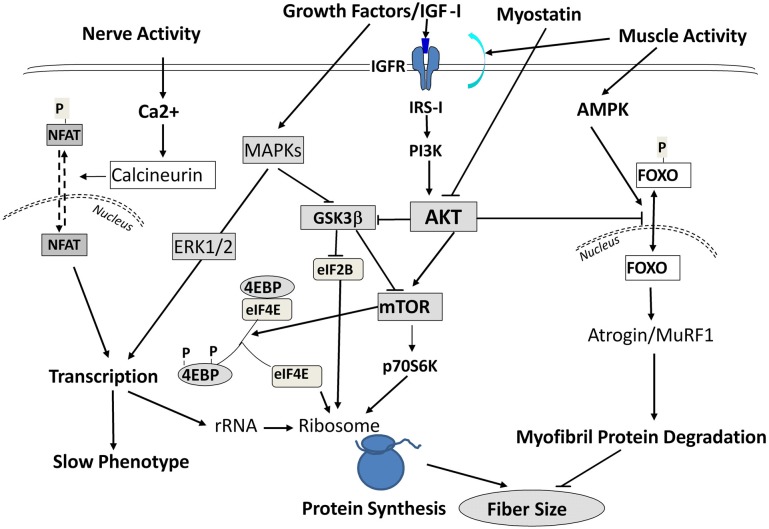
**Signaling Pathways Leading to Altered Protein Balance Affecting Muscle Fiber Size**. A simplified schematic of signaling pathways affecting protein balance in muscle fibers. Signals are initiated by either growth factors, nerve, or muscle contractile activity and are transmitted into the cells to affect protein synthesis via mTOR/Akt signals, protein transcription via MAPK ERKs, and protein degradation through Foxo/Atrogin/Murf1 action. For further information, the readers are referred to excellent reviews by Sandri (2008); Elkina et al. (2011); Bonaldo and Sandri (2013) and by Frost and Lang (2012).

Interestingly, during SI these above responses occurred in the absence of any functional up-regulation of key translational regulatory proteins (e.g., p70 S 6 kinase, and eukaryotic 4E binding protein-1) that are normally necessary for augmenting protein translation processes (Figure 5) to compensate for the decreased “protein translational capacity” noted above. Therefore, these observations collectively demonstrate that (1) the molecular changes accompanying SI-induced muscle atrophy are not necessarily the reverse of those alterations normally occurring during muscle hypertrophy in response to anabolic cascades; and (2) the rapid and marked atrophy that defines the SI model of neuromuscular inactivity is likely the result of multi-factorial processes negatively affecting transcriptional, translational, and post-translational processes, the latter of which enhances net protein degradation targeting the myofibril/sarcomeric complex (Figure 5).

### Hindlimb suspension model

As noted above, ~90% of the fibers in the rat soleus muscle express the slow-type I MHC protein (Giger et al., 2000). HS induces the MHC isoform population to shift from a slow type I MHC predominance toward a predominance of the type II MHC isoforms (Caiozzo et al., 1997). Thus, we hypothesized that this shift in expression involving the slow type I MHC down regulation is transcriptionally regulated through specific cis-elements in the type I MHC promoter.

In weight-bearing rats, the relative luciferase activity of the longest type I MHC promoter fragment (−3500 bp) is three-fold greater than the shorter promoter constructs, suggesting that an enhancer sequence is present in the up stream promoter region(Giger et al., 2000). After 1 week of HS, the reporter activities of the −3500, −914, and −408-bp constructs were significantly reduced by ~40% as compared to the control muscles (Giger et al., 2000). However, no differences in −215 bp promoter activity were observed between HS and weight bearing, control muscles. These findings suggested that (1) there are key elements in the type I promoter in the −408 sequence that confer activity of the type I MHC gene, and (2) transcriptional events are pivotal to the altered expression of the type I MHC gene in response to unloading stimuli induced by HS.

In a follow up study, a similar paradigm was conducted to ascertain the key sequences responsible for the transcriptional alterations that occur in response to HS (Giger et al., 2004). This study utilized mutation analyses involving six putative regulatory elements within the −408 promoter sequence. These experiments demonstrated that three elements, i.e., an A/T rich sequence, a proximal muscle-type CAT (βe3) sequence, and an Ebox (−63 bp) sequence likely interact to regulate the basal level of the slow, type I MHC promoter in normal control soleus muscle; and these elements function collectively as an “unloading response sequence”. Gel mobility shift assays revealed a diminished level of complex formation involving the βe3 and E-box probes using nuclear extract obtained from soleus muscles of HS rats when compared to the soleus from control rats. Super-shift assays indicated that transcriptional enhancer factor 1 (TEF-1) and myogenin factors bind the βe3 and E-box probes, respectively in control soleus. Western blots showed that the relative concentrations of TEF-1 and myogenin factors were significantly attenuated in the unloaded soleus compared with the normal control muscle. These observations suggest that the down regulation of the slow type I MHC in response to unloading is due, in part, to a significant decrease in the level of expression of these transcription factors being available for binding to the positive regulatory elements.

**Figure 6:**
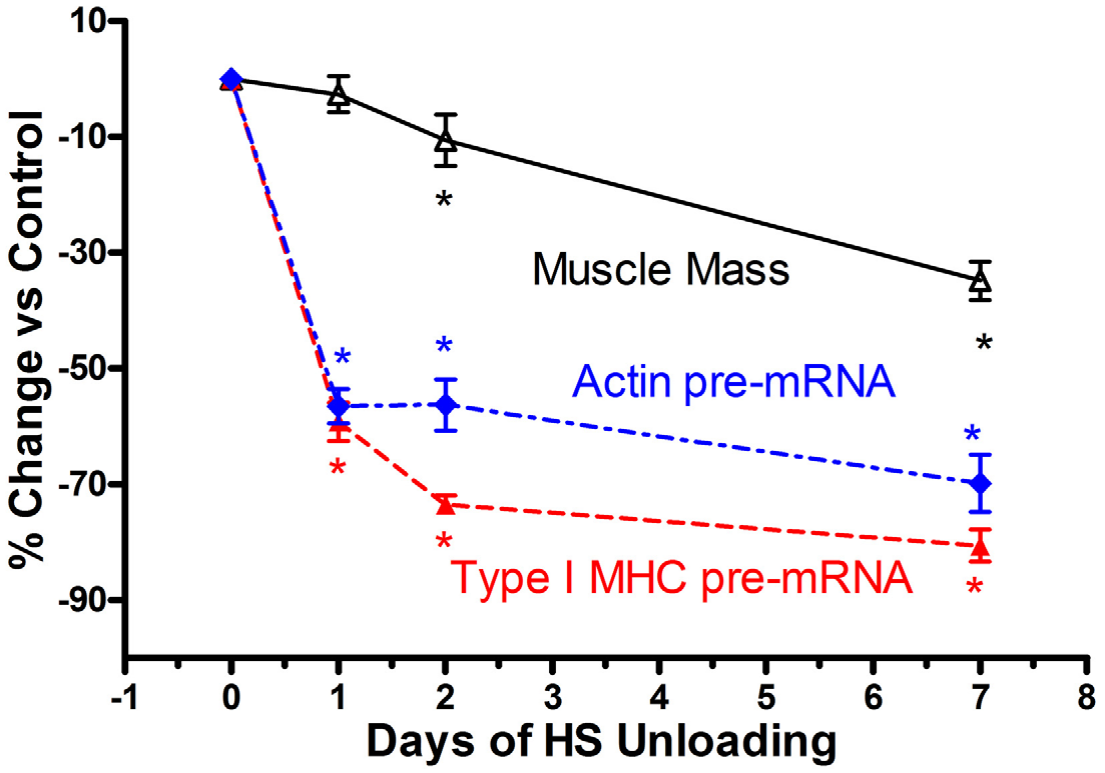
**Muscle atrophy in antigravity muscle is rapid and preceded by transcriptional repression of myosin and actin**. Change in soleus mass, actin and type I MHC pre-mRNA in response to 7 days hindlimb suspension (HS) unloading. ^*^ is for *p* < 0.05 vs. 0 Day time point for each variable.

Based on the above findings implicating transcriptional inhibition of the transfected slow type I MHC promoter in response to a short duration HS paradigm (i.e., 7 days), we hypothesized that the *in vivo* type I MHC promoter must undergo an early response to unloading stimuli (Giger et al., 2009). Given the fact that α-actin (acta-1) comprises ~40% of the myofibril protein milieux and undergoes altered expression in response to paradigms impacting muscle mass (Carson et al., 1996), we further postulated that skeletal actin-1 is a primary player during the atrophy process in the soleus muscle during states of unloading. Therefore, we characterized the dynamic changes in the unloaded soleus muscle, *in vivo*, following short bouts (1, 2, and 7 days) of HS (Figure 6), testing the hypothesis that transcriptional events respond within several hours after the initiation of the atrophic stimulus. In fact, we observed that after only 1 day of HS, the primary transcript levels of skeletal acta-1 and type I MHC pre-mRNA were significantly reduced by more than 50% compared to weight-bearing control rats. The degree of the decline for the mRNA expression of actin and of type I MHC lagged behind that of the pre-mRNA after 1 day of HS, but large decreases were observed after 2 and 7 days of HS. Although the faster MHC isoforms, IIx and IIb, began to be expressed in the soleus muscle after 1 day of HS, a relatively significant shift in mRNA expression from the slow MHC isoform type I toward these fast type II MHC isoforms did not emerge until ~7 days of HS. Interestingly, 1 day of HS was sufficient to show significant decreases in mRNA levels of putative signaling factors such as serum response factor (SRF), suppressor of cytokine signaling-3 (SOCS3) and striated muscle activator of Rho signaling (STARS); whereas the decreases in transcriptional enhancing factor 1 (TEF-1), yin yang-1(YY1) were less robust. The protein level of actin and type I MHC were significantly decreased after 2 days of HS, whereas, SRF protein was markedly decreased only after 7 days of HS. Thus, our results show that after only 1 day of unloading, pre-mRNA and mature mRNA expression of muscle proteins and muscle-specific signaling factors are significantly reduced. These findings suggest that the down regulation of the synthesis side of the protein balance equation that occurs in atrophying muscle is initiated rapidly during the unloading stimulus cascade, especially when one considers the observation that total/ribosomal RNA concentration also is reduced early on in the unloading stimuli (Giger et al., 2009).

## Historical perspective concerning the impact of unloading on muscle mass and contractile phenotype

Historically, the dogma concerning unloading stimuli impacting muscle atrophy has centered on the notion that both a decrease in protein synthesis and an increase in protein degradation account for the net muscle loss/atrophy. Booth et al., to our knowledge, were the first investigators to systematically examine the role of protein synthesis and pretranslational markers (MHC and actin gene regulation) on muscle atrophy processes using rodent limb immobilization models (Watson et al., 1984; Babij and Booth, 1988). By focusing on the fast twitch gastrocnemius muscle, their findings suggested that protein synthesis deficits were early contributors to the muscle atrophy during the early stages of immobilization. More recently, factors regulating catabolic processes such as the FOXO-ubiquitin/atrogin-related cascade have been shown to play a pivotal role in the protein degradation process (Sandri et al., 2004). In the context of the above observations, which for the most part, have focused primarily on translational/post-translational events (Sandri et al., 2004; Glass, 2005; Baar et al., 2006; Kandarian and Jackman, 2006), it appears that transcriptional-pretranslational events have received less attention. In fact, experiments performed by Booth's group (Watson et al., 1984; Babij and Booth, 1988) indicated that actin or type I MHC mRNA expression contributed little to the atrophy response until approximately seven or more days had elapsed in either limb immobilization or HS paradigms. However, our findings, as noted above for both the SI and HS models, clearly document that marked rapid losses of MHC and actin, pre- mRNA, MHC, and actin mRNA, and total ribosomal RNA collectively play a significant role in the context of protein balance being biased to a net degradation of the myofibril apparatus. As shown in Figure 6, which is a compilation of data derived from the recent paper by Giger et al. (2009), it is obvious that transcription of both the slow-type I MHC and actin genes are repressed within 1 day of HS, well before there are significant alterations in muscle mass. These findings clearly suggest that transcriptional/pretranslational processes are playing a significant role in the early stages of unloading. Given the additional observation that ribosomal RNA, the building block for protein translation, is significantly reduced at the same time, it is obvious that these combined alterations play a key role in the remodeling of antigravity muscle in response to unloading.

## Mechanisms of slow to fast MHC gene switching during unloading: role of chronic low-frequency electrical stimulation

The nerve activity patterns are thought to regulate MHC gene expression and muscle fiber phenotype. It has been proposed that skeletal muscles expressing predominantly the slow such as the soleus, type I MHC isoform are regulated by a stimulation profile consisting of a slower frequency stimulation paradigm (Henning and Lomo, 1985). For example, in hind limb unloading, chronic low frequency stimulation of 20 Hz proved to be effective in maintaining the slow fiber-type without having any impact on preserving muscle mass (Leterme and Falempin, 1994; Dupont et al., 2011). These interesting observations suggest that in order to maintain overall muscle mass in the face of unloading paradigms stimuli that resemble gravity loading forces are necessary to maintaining muscle mass.

## Mechanisms of slow to fast MHC gene switching during unloading: role of non-coding antisense RNA

**Figure 7:**
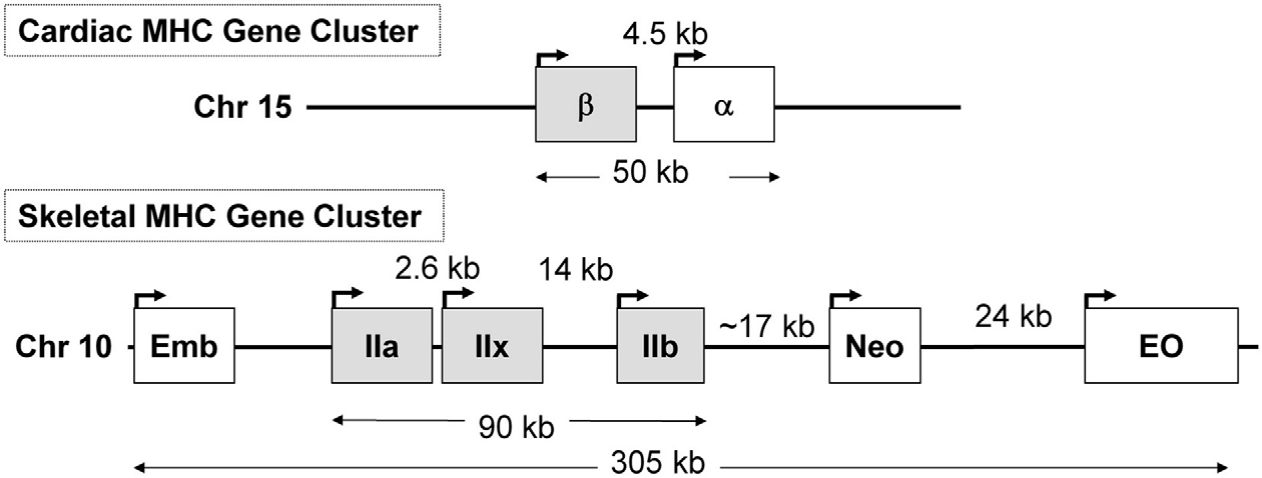
**The organization of the sarcomeric myosin heavy chain (MHC) gene family**. At least 8 MHC genes are expressed in striated muscle and are found in two clusters: (1) the cardiac MHC gene cluster on rat chromosome 15, which consists of the type I also called β and the α cardiac MHC genes. Type I is the slow MHC expressed in slow skeletal muscle fibers; (2) the skeletal MHC gene cluster on rat chromosome 10, the embryonic (Emb), fast IIa, IIx, IIb, neonatal (Neo) and extraocular (Eo) genes are located in tandem in the order depicted. This MHC gene organization, order, head to tail orientation, and spacing has been conserved through millions of years of evolution and could be of great significance to the way these genes are regulated in response to various stimuli. Human and mouse cardiac MHCs are found on chromosome 14; whereas human skeletal MHCs are found on chromosome 17, and the mouse skeletal MHCs are found on chromosome 11.

In previous sections of this review, we described that during SI there was a switching of MHC gene expression whereby both the slow-type I and fast type IIa genes were transcriptionally repressed while the faster type IIx and to a lesser extent the type IIb MHC genes were expressed *de novo* in the unloaded SI soleus muscle (Huey et al., 2001, 2002). In the context of these observations it is important to point out the unique genomic organization of the MHC gene family in mammals. This gene family comprise at least eight members: two cardiac MHC genes alpha and beta (I), three adult fast MHC (IIa, IIx, and IIb), two developmental MHC (Embryonic and Neonatal), and one specialized form the extraocular (EO) MHC gene. Note that cardiac beta is the same as the type I MHC gene that is expressed in slow skeletal muscle fibers. These MHC genes are clustered into two clusters: (1) the cardiac MHC on chromosome 15 in the rat, and (2) the skeletal MHC cluster on chromosome 10 (Figure 7). These genes' clustering, orientation, and tandem organization have been conserved through millions of years of mammalian evolution. This conserved configuration raises questions as to whether this particular MHC gene alignment is of functional significance in their patterns of regulation.

Recent evidence has implicated a non-coding antisense RNA in the coordinated regulation of two positioned genes in tandem, which emphasize the importance of the genomic organization of these MHC genes in their coordinated regulation. For example, in 2003 Haddad et al. (2003c) reported the novel discovery that in cardiac muscle, a naturally occurring antisense RNA to the cardiac β (type I) MHC gene is involved in cardiac MHC gene regulation. Cardiac α and β MHC isoforms are the products of two distinct genes that are organized in tandem in a head to tail position on the same chromosome in the order of β → α (Figure 7); and are separated by a ~4.5 kb intergenic space. A long non-coding antisense RNA is transcribed from the DNA strand that is opposite to the MHC genes creating a β antisense RNA. This antisense-β transcript was implicated with the MHC isoform gene switching in the heart in response to both diabetes and hypothyroidism (Haddad et al., 2003c). Given these observations, studies were subsequently carried out on skeletal muscle to ascertain if non-coding antisense RNA expression in slow and fast skeletal muscle contributes to the patterns of MHC gene expression in response to unloading stimuli.

**Figure 8:**
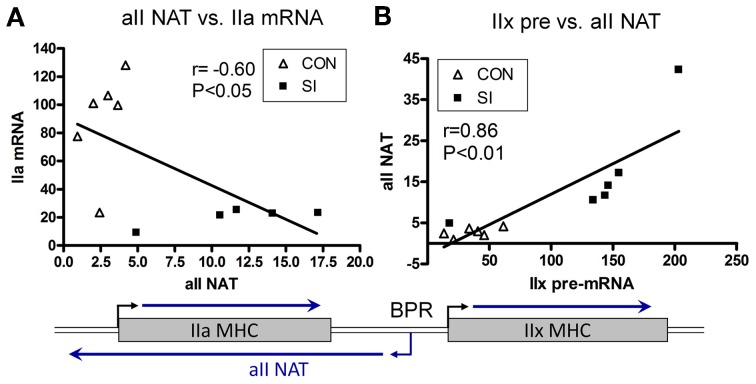
**Relationship between Sense and Antisense Transcripts in Soleus Muscle**. IIa mRNA is inversely proportional to IIa NAT **(A)**; whereas IIx pre-mRNA is correlated positively to IIa NAT **(B)**. Lines are generated by regression analyses (GraphPad Prism). r, Pearson coefficient determined with correlation analyses for each set. Open triangle, control; closed square, SI. Also shown is a schematic of the IIa and IIx genes, and transcriptional activity in the intergenic region depicting a bidirectional promoter (BPR) in the IIx 5′ proximal region (Pandorf et al., 2006).

In 2006, Pandorf et al. (2006) published a paper which investigated type II MHC gene regulation in the slow type I soleus muscle fibers undergoing a slow to fast MHC transformation in response to seven days of SI. Transcriptional products were examined of both the sense and antisense strands across the IIa-IIx-IIb MHC gene locus presented in Figure 7. Results showed that the mRNA and pre-mRNA of each MHC had a similar response to the SI stimulus, suggesting regulation of these genes at the transcriptional level. In addition, detection of a previously unknown antisense strand transcription occurred that produced *natural antisense transcripts* (NATs). RT-PCR mapping of the RNA products revealed that the antisense activity resulted in the formation of three major products: aII, xII, and bII NATs, i.e., antisense products of the IIa, IIx, and IIb genes, respectively. The key observation of this experiment was that the SI-induced inactivity caused a marked inhibition of both the slow type I and type IIa genes along with up regulation of both the IIx and IIb genes. Thus, the inactivity model of SI (1) negatively impacts transcription of the type I MHC gene directly by inhibiting its promoter (see above), and (2) induces anti sense aII NATs that primarily repress transcription of the IIa MHC gene (Figure 8), thereby creating a switch from slow I/IIa to a fast IIx fiber of the normally slow soleus muscle Importantly, this observation explains the existence of type I/IIx hybrid fibers reported previously by Caiozzo et al. (1998); and nulling out the transition schemes originally proposed by Pette and Staron, that MHC transitions in muscle fibers occur in a precise order I↔IIa↔IIx↔IIb (Pette and Staron, 1990).

## Mechanisms of slow to fast MHC gene switching during unloading: role of epigenetic modification of histones at MHC genes

**Figure 9:**
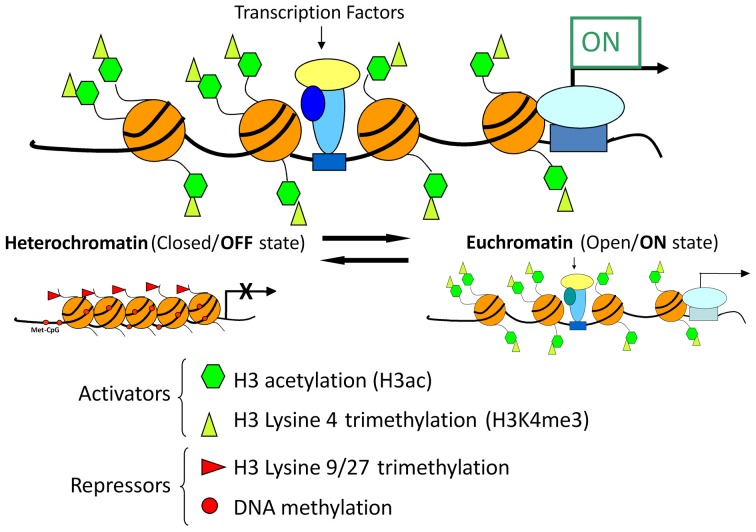
**Chromatin state and gene transcription**. Model for chromatin factors interacting with transcription factors to regulate transcription of a gene. Histone modifications and DNA methylation are important factors in regulating the chromatin from active to repressed and vice versa. Histone H3 acetylation and histone H3 methylation and lysine 4, are both associated with an active chromatin state. In contrast, histone H3 methylation at lysine 9 or lysine 27 as well as DNA methylation are associated with repressive chromatin state. Chromatin is in a dynamic equilibrium between the two states.

Recent advances in chromatin biology have advanced our understanding of gene regulation suggesting that alterations in gene regulation are highly dependent upon post-translational modifications to the histones, which package genes in the nucleus of cells. Active genes are known to be associated with acetylation of histones (H3ac) and trimethylation of lysine 4 in histone H3 (H3K4me3) as presented in Figure 9. In 2009 our group headed by Clay Pandorf used the chromatin immunoprecipitation (ChIP) technique to examine histone modifications at the MHC genes expressed in fast vs. slow fiber-type muscles using the model of HS, which induces a shift to fast MHC genes expression in the slow, type I soleus muscle (Pandorf et al., 2009). The findings indicate that both H3ac and H3K4me3 varied with the transcriptional activity of the MHC in fast fiber type plantaris and slow fiber type soleus muscles. During MHC transitions with muscle unloading, histone H3 at the type I MHC gene becomes de-acetylated in correspondence with down regulation of that gene, while up regulation of the fast type IIx and IIb MHCs occurs in conjunction with enhanced H3ac in those MHC genes. Enrichment of H3K4me3 is also increased at the type IIx and IIb MHCs when these genes are induced by unloading stimuli. Down regulation of the IIa MHC gene, however, was not associated with a corresponding loss of H3ac or H3K4me3. These observations demonstrated the feasibility of using the ChIP assay to understand the native chromatin environment in adult skeletal muscle, and further suggest that the transcriptional state of types I, IIx, and IIb MHC genes are sensitive to histone modifications both in different muscle fiber types and in response to altered loading states. Additional studies are needed to ascertain the temporal nature of alterations in the histone machinery relative to the alterations in transcriptional activity the target gene's promoter in response to altered loading state. These results demonstrate the important role of histone biology in understanding the plasticity of skeletal muscle under different activity/inactivity paradigms.

## Strategies to ameliorate muscle atrophy during early and extended stages of muscle unloading

### Hindlimb suspension

In previous sections, we pointed out the rapid nature of unloading-induced deficits in transcriptional/pretranslational activity of key marker myofibril genes such as slow MHC and actin (Giger et al., 2000). In addressing this topic, we postulated that to either ameliorate and/or prevent such deficits one needed to (1) employ high RE stimuli at the outset of initiating the unloading paradigm; and (2) utilize a RE paradigm that was effective in stimulating muscle hypertrophy in ambulatory rats. Therefore, we utilized an isometric paradigm to blunt the atrophy response during the first 5 days of HS in which there is a rapid unloaded stimulus occurring (Haddad et al., 2006). The findings of this study showed that (1) there was a ~20% decrease in absolute muscle mass; (2) the normalized myofibril fraction concentration and content were decreased; and (3) a robust isometric training paradigm known to induce a hypertrophy response, failed to maintain the myofibril protein content. This response occurred despite fully blunting the increases in the mRNAs for atrogin-1, MURF-1, and myostatin, i.e., markers of an activated catabolic state (Figure 5). Analyses of the IRS-1/PI3K/AKT markers indicated that the abundance of IRS-1 and the phosphorylation state of AKT and p70S6K were decreased relative to the normal control state. Thus the resistance training failed to maintain these anabolic signaling markers at an appropriate regulatory level.

Therefore, Adams et al. (2007) initiated an additional study to determine if RE involving a greater contractile volume of loading per each training session (3 s per contraction) along with integrating isometric, concentric, and eccentric actions during each contraction, would be effective in preventing unloading-induced muscle atrophy (Adams et al., 2004). Rats were exposed to 5 days of muscle unloading via HS. During each session, one leg received electrically stimulated RE and the contra lateral leg served as the control. The results indicated that the combined mode of RE provided an effective anabolic stimulus to maintain both muscle mass and myofibril content of the trained relative to the contra lateral control muscle. Relative to the control muscle, the RE stimulus also increased the levels of total RNA (indicative of translational capacity) enhanced mRNA levels for several anabolic/myogenic markers such as IGF-I, myogenin, myoferlin, and procollagen III-α-1; and decreased the mRNA levels of myostatin, a key negative regulator of muscle fiber size (Elkina et al., 2011). The combined mode RE protocol also increased the activity of anabolic signaling regulators such as p70S6 kinase and hyperphoso-4E-binding protein. Collectively, these results indicate that a combination of static- and dynamic-mode RE of sufficient volume provides an effective paradigm to enhance anabolic/myogenic mechanisms to counteract the initial stages of the unloading-induced muscle atrophy cascade.

### Spinal isolation

Kim et al. (2008) examined anabolic and catabolic markers of muscle protein metabolism in SI-induced atrophying muscles with and without daily short-duration, high resistance isometric contractions. The stimulation protocol consisted of pulses (100 Hz, 4 s duration) delivered once every 30 s for 5 min, followed by 5 min of rest, repeated three times: this entire sequence was repeated twice per day with a 9-h rest interval). The total amount of activation was 4 min per day for 5 consecutive days. Adult rats were assigned to either a normal control or SI group in which one limb was stimulated (SI-Stim) with brief bouts of high-load isometric contractions (via a microstimulator provided by the Alfred Mann Foundation implanted parallel to the sciatic nerve) and one limb not stimulated (SI-C). Both the MG and soleus weights (relative to body weight) in the SI-C were atrophied by ~30%, but were maintained at control levels in the SI-Stim group. Activity of the IGF-I/PI3K/AKT pathway of protein anabolism was similar among all groups in the MG. Expression of atrogin-1 and muscle RING finger-1(MURF-1) markers were higher in the MG and soleus of the SI vs. the normal control group, and were maintained at control level in the SI-Stim group. Compared with the control state, myostatin, an anti-growth factor, was unaffected in the MG and soleus in the SI control group, but was lower in the MG of the SI-Stim group. These results demonstrated that up regulation of specific protein catabolic pathways play a critical role in SI-induced atrophy; whereas, this response was blunted by 4 min of daily high-resistance electromechanical stimulation, and was able to preserve most of the muscle mass. Although the protein anabolic pathway (IGF-1/PI3K/AKT) appears to play a minor role in regulating muscle mass in the SI Model, increased “translational capacity” via increases in total ribosomal RNA may have contributed to muscle mass preservation in response to isometric contractions as appears to occur in the HS model described above (Adams et al., 2007).

A more prolonged parallel study then was performed to determine the effects of chronic inactivity on the catabolic and anabolic pathways in a slow (soleus) and fast (plantaris) muscle (Kim et al., 2010). The stimulation protocol consisted of pulses (100 Hz, 1 s duration) delivered once every 30 s for 5 min, followed by 5 min of rest, repeated six times consecutively (Stim 1) or with a 9-h interval after the third bout (SI-Stim 2). The total amount of activation was 1 min per day for 30 consecutive days. The SI-Stim1 and SI-Stim2 paradigms attenuated plantaris muscle loss by 20 and 38%, respectively, whereas, SI-Stim 2 blunted soleus atrophy (24%) relative to SI-C. Muscle mass alterations occurred independently of the IGF-1/PI3K/AKT pathway. No relationships between SI or electro-mechanical stimulation and expression mechanisms of several atrophy markers were altered. These particular data suggest that regulatory mechanisms for maintaining muscle mass previously shown in more acute states of atrophy (early stages of atrophy) noted above differ substantially from those situations occurring during “chronic states” of long term atrophy. Clearly, more research is needed in this important area, because the initiation of countermeasure programs after the atrophy processes is underway appears to be more challenging than immediately initiating the counter measure before the atrophy is well primed.

## Summary and future directions

In this review we have examined three models of unloading the hindlimb skeletal muscles of rodents, i.e., spaceflight/microgravity, a ground based HS model, and the unique model of SI. All three models markedly decrease the frequency and magnitude of ground reaction forces. However, the former two models still enable movement of the hindlimbs; whereas the SI model silences both neuromuscular activation/loading and movement. These models clearly show similar qualitative and quantitative alterations resulting in muscle fiber atrophy along with a marked slow to fast shift in the fiber contractile phenotype. This latter alteration is specifically linked to altered gene expression of the MHC gene family of motor proteins, especially the slow-type I MHC along with altered actin expression, both of which account for the major composition of the myofibril fraction of the muscle. Transcriptional, pretranslational, translational, and post-translational events spearhead the muscle atrophy processes resulting in a marked decrease of the myofibril fraction, which contains the contractile machinery. Although these subcellular alterations interact to create a rapid reduction in skeletal muscle mass, especially in those muscles expressing primarily slow motor units, it is important to point out that transcriptional events, along with ribosomal RNA levels, are primary events that catalyze the rapid phase of the atrophy process. On the other hand, the events that cause a shift in slow to fast MHC gene expression depend upon unique regulatory processes involving mechanisms associated with non-coding antisense RNA expression processes to inhibit expression of the “slower fast” isoform genes, i.e., type IIa MHC, to enable up regulation of the faster type IIx and IIb isoforms. In addition, new findings suggest that epigenetic mechanisms impacting histone modifications are also occurring and may be a key coordinator of this gene switching process. For example, histone modifications occurring in response to altered loading states may be regulated by antisense transcription of the MHC genes. New research will be needed to test the role of antisense non-coding RNA in recruiting histone modifying enzymes as well as other factors capable of altering chromatin function and gene transcription.

Epigenetic regulation of gene transcription is complex; it involves chromatin structure and requires interaction and cooperation among several histone modifying enzymes, remodeling enzymes, and transcription factors. Another epigenetic phenomenon is DNA methylation. This process is more dynamic than previously thought. It is altered in response to loading stimuli and in turn, it alters gene function (Barrès et al., 2012). Very little is known about the role of the DNA methylation in MHC gene regulation Future research in these areas is needed to expand our understanding of the complex multilayered regulation of the muscle genes that are responsible to muscle fiber diversity.

In spite of a clearer understanding of the molecular mechanisms underlying muscle plasticity, atrophy, and contractile phenotype switching, our understanding of the mechanical and metabolic events that trigger these adaptive events are minimal. For example the obvious differences in the sensitivity of different muscle types to the “unloading” perturbations addressed in this review have received almost no attention. Another event in the atrophic process that has received remarkably little focus has been how such a rapid disassembly of the highly structural organized contractile protein can occur in a muscle such as the soleus, and yet, the muscle remains functional. Finally, the role of the nervous system, particularly the motoneuron, in being a source of regulation via activity-independent (neurotrophic) and/or activity-dependent (mechanical) mechanisms, of the molecular events discussed herein remains poorly understood.

What is clear from current research is that gene regulation is a multilayered process with many molecular layers interacting together to achieve the fine control. Large gaps exist in our understanding of molecular processes underlying muscle function and the response to perturbation. Advances in the area of functional genomics, proteomics, and metabolomics hold the promise to broaden our understanding of muscle plasticity and can potentially uncover novel regulatory pathways that are involved in muscle adaptation to patho-physiological processes as manifest in altered muscle mass and muscle function. Delineating interactions among various regulatory layers will be essential for our full understanding of these complex processes, and will eventually lead to specific targets for intervention against muscle atrophy and fiber-type shifts.

### Conflict of interest statement

The authors declare that the research was conducted in the absence of any commercial or financial relationships that could be construed as a potential conflict of interest.
